# Early regional cerebral grey matter damage predicts long-term cognitive impairment phenotypes in multiple sclerosis: a 20-year study

**DOI:** 10.1093/braincomms/fcae355

**Published:** 2024-10-12

**Authors:** Stefano Ziccardi, Francesco Crescenzo, Maddalena Guandalini, Gulser Caliskan, Luigi Martinelli, Agnese Tamanti, Gian Marco Schiavi, Albulena Bajrami, Damiano Marastoni, Massimiliano Calabrese

**Affiliations:** Neurology Section, Department of Neurosciences, Biomedicine and Movement Sciences, University of Verona, 37134 Verona, Italy; Neurology Unit, Mater Salutis Hospital, 37045 Verona, Italy; Neurology Section, Department of Neurosciences, Biomedicine and Movement Sciences, University of Verona, 37134 Verona, Italy; Epidemiology and Medical Statistics Section, Department of Diagnostics and Public Health, University of Verona, 37134 Verona, Italy; Epidemiology and Medical Statistics Section, Department of Diagnostics and Public Health, University of Verona, 37134 Verona, Italy; Neurology Section, Department of Neurosciences, Biomedicine and Movement Sciences, University of Verona, 37134 Verona, Italy; Neurology Section, Department of Neurosciences, Biomedicine and Movement Sciences, University of Verona, 37134 Verona, Italy; Neurology Section, Department of Neurosciences, Biomedicine and Movement Sciences, University of Verona, 37134 Verona, Italy; Neurology Section, Department of Neurosciences, Biomedicine and Movement Sciences, University of Verona, 37134 Verona, Italy; Neurology Section, Department of Neurosciences, Biomedicine and Movement Sciences, University of Verona, 37134 Verona, Italy

**Keywords:** multiple sclerosis, cognitive impairment, regional atrophy

## Abstract

Despite grey matter atrophy in cortical and subcortical regions has been related to cognitive impairment in multiple sclerosis, only a few studies evaluated its predictive value for alterations in the long-term. We aimed to determine early predictors of cognitive status after 20 years of multiple sclerosis. In this longitudinal retrospective study, participants underwent a 1.5 T MRI scanning at diagnosis (T0) and after two years (T2), which included the evaluation of regional grey matter volume loss patterns. All individuals with multiple sclerosis underwent a comprehensive neuropsychological assessment at the end of the study and were classified considering their global and specific cognitive domains status (memory, attention/information processing speed, executive functioning). Clinical and MRI characteristics were assessed as predictors of long-term cognitive impairment. Analysis of covariance, *t*-test, unadjusted and adjusted (for age, sex, disease duration, volume of white matter lesions, volume of cortical lesions) logistic regression were conducted. One hundred seventy-five people with multiple sclerosis (118 females; mean ± SD age at the end of study = 47.7 ± 9.4 years) clinically followed for 20 years from onset (mean ± SD = 19.9 ± 5.1) were evaluated. At the end of the study, 81 (47%) were classified as cognitively impaired: 38 as mildly impaired (22%), and 43 as severely impaired (25%). In particular, 46 were impaired in memory (27%), 66 were impaired in attention/information processing speed (38%), and 71 were impaired in executive functioning (41%). Regression models identified precuneus (adjusted odds ratio = 3.37; *P* < 0.001), insula (adjusted odds ratio = 2.33; *P* = 0.036), parahippocampal gyrus (adjusted odds ratio = 2.07; *P* < 0.001) and cingulate (adjusted odds ratio = 1.81; *P* = 0.009) as the most associated regions with global cognitive impairment and domains-specific cognitive alterations after a mean of 20 years of multiple sclerosis, after adjusting for demographic and clinical variables as well as for focal white matter and grey matter damage. Early grey matter volume loss of specific cortical and deep grey matter regions predicts global and domain cognitive alterations after 20 years from multiple sclerosis diagnosis.

## Teaser

In the present study including 175 patients followed for 20 years, Ziccardi *et al*. report that neurodegenerative processes are present since the time of multiple sclerosis diagnosis: early cortical damage in specific brain regions seems a reliable predictive marker of long-term global and domain-specific profiles of cognitive impairment.

## Graphical Abstract

**Graphical Abstract:**
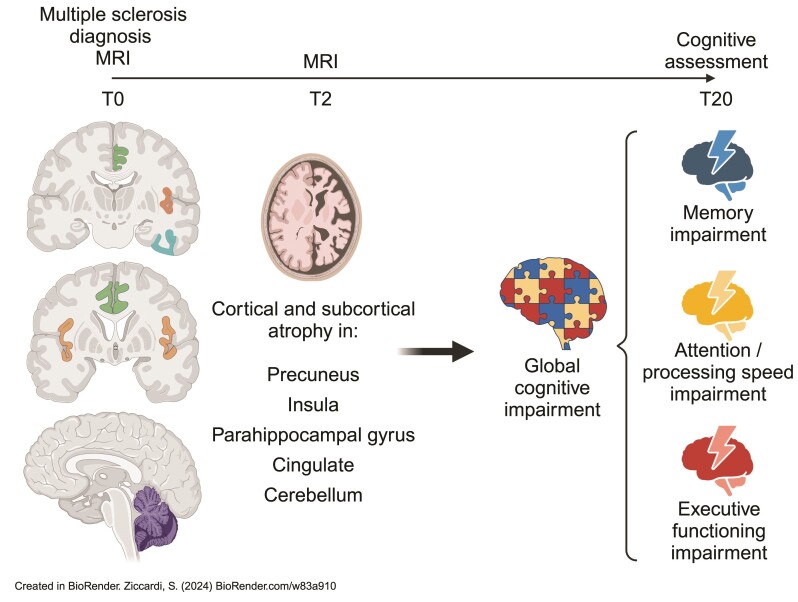

## Introduction

Multiple sclerosis is the most frequent cause of non-traumatic neurological disability among young adults. Focal (cortical demyelinating lesions) and diffuse (cortical atrophy) grey matter (GM) damage occurs in multiple sclerosis^1^ since the earliest stages of the disease^2^ and progresses over time.^3^ Cortical atrophy and subcortical volume loss across the brain are not uniform:^4,5^ some areas, such as frontotemporal areas, hippocampus, cerebellum and thalamus, are more susceptible than others to neurodegeneration.^6,7^

In addition to physical disability, cognitive impairment (CI) is highly frequent in people with multiple sclerosis, affecting up to 65% of the individuals, depending on classification criteria and disease course.^8^ CI can occur even in a preclinical phase and tends to worsen over time, dramatically impacting on the quality of life of patients and their caregivers.^9^ Mirroring what happens for brain atrophy, not all cognitive functions are equally involved: memory, information processing speed, attention and executive functions are the most affected domains in multiple sclerosis.^10^ CI patients tend to develop a poorer long-term prognosis in terms of physical disability and mortality.^11,12^

Moreover, growing evidence highlighted the association between CI and GM damage:^11,13,14^ several cross-sectional studies described that CI patients showed a more pronounced cortical and deep GM atrophy, particularly in the frontal and temporoparietal regions, the cerebellum and in the thalamus, hippocampus, insula and cingulate.^15-18^ Furthermore, some longitudinal studies (between 5 and 13 years of follow-up evaluation) showed the relationship between CI and cortical damage, even based on global GM atrophy measures or single cognitive tests/brief neuropsychological batteries.^19-21^

Nevertheless, the ability to predict the evolution of cognitive disability along the multiple sclerosis course and the identification of factors contributing to the individual risk of long-term cognitive impairment accumulation remains an unmet need.^22,23^ GM damage has been identified to be a better predictor of cognitive impairment than multifocal white matter damage,^20^ however the debate still represents a hot topic. Recently, a retrospective longitudinal study highlighted that cortical damage at diagnosis, in terms of cortical lesions burden, represents a predictive marker for long-term CI.^24^ People with multiple sclerosis who showed focal cortical damage since diagnosis have a significantly higher probability of developing severe cognitive alterations after 20 years of multiple sclerosis.^24^

In addition to the abovementioned studies and the actual knowledge about the relationship between GM damage and cognitive status, we herein aimed to shed light on the predictive role of the cortical damage (in terms of global and regional GM volume loss) accumulated in the first two years from the multiple sclerosis diagnosis with reference to long-term global CI and phenotypes of cognitive alterations, to provide a fact-finding picture with a longitudinal perspective of the disease evolution over a long follow-up time.

## Materials and methods

### Study design and participants

In this retrospective longitudinal study, 175 individuals with multiple sclerosis were enrolled from a cohort presently followed at the Multiple Sclerosis Centre of the Veneto Region (Verona, Italy), having an average of 20 years of clinical follow-up since the clinical onset [mean ± standard deviation (SD) = 19.9 ± 5.1 years]. One hundred seventy individuals have been previously reported in a study on focal damage predictors of global cognitive status;^24^ in contrast, here we focused on brain global/regional GM volumes as predictors of long-term global cognitive status and domain cognitive functioning. Inclusion criteria were: (i) diagnosis of relapsing-remitting multiple sclerosis (RRMS) or secondary progressive multiple sclerosis (SPMS) at the end of the 20-years follow-up period study (end of study, EOS) according to the most recent diagnostic criteria;^25^ (ii) availability of MRI examination including the 3D volumetric T1 sequence performed at the time of diagnosis (T0) and two years after from the baseline MRI (T2); (iii) longitudinal clinical follow-up performed by a neurologist having an extensive experience of multiple sclerosis (M.C.), inclusive of the Expanded Disability Status Scale (EDSS) assessment;^26^ and (iv) comprehensive neuropsychological assessment performed at the EOS. Exclusion criteria were: (i) the presence of any concomitant neurological condition (other than multiple sclerosis); (ii) the presence of any psychiatric or other pathological conditions; (iii) substance abuse; and (iv) hearing impairment, severe upper limb impairment and visual disorders that could interfere with cognitive performance.

The study was approved by the local Ethics Committee and was conducted in accordance with the Declaration of Helsinki (2013). Patients gave informed consent.

### Neuropsychological assessment

At the EOS, all patients underwent a comprehensive neuropsychological assessment in a clinically stable phase (no relapses/acute corticosteroid treatments in the 30 days before) by using the Brief Repeatable Battery of Neuropsychological Tests (BRB-NT),^27^ the Stroop Test (ST),^28^ the Phonemic Semantic and Alternate Verbal Fluency Test (VF),^29^ and the Modified Five Point Test (MFPT).^30^ The BRB-NT is composed of: the Selective Reminding Test (SRT), a test of verbal learning and delayed memory recall; the 10/36 Spatial Recall Test (SPART), a test of visuospatial learning and delayed memory recall; the Symbol Digit Modalities Test (SDMT), a test of visual information processing speed and attention; the Paced Auditory Serial Addition Task (PASAT-3 and PASAT-2), a test of attention, calculation and auditory information processing speed; and the Word List Generation (WLG), a test of semantic verbal fluency with a double category. The ST was conducted to assess attention and automatic response inhibition. The VF is a test of phonemic and semantic fluency, as well as the switching between the two in the alternate version of the test. The MFPT assesses figurative fluency, use of strategies and error monitoring.

Raw scores were adjusted for age, sex and education according to the normative data of each specific test: adjusted scores lower than the fifth percentile (cut-off) were considered as failed. People with multiple sclerosis were classified into three groups based on their scores on all the cognitive tests administered (BRB-NT, ST, VF, MFPT), using an approach widely used and recommended in literature: ‘severely cognitive impaired’ (sCI) for patients with *Z*-scores ≤ −2 on at least two different cognitive domains of the neuropsychological tests, ‘mildly cognitive impaired’ (mCI) for patients with *Z*-scores ≤ −1.5 on at least two different cognitive domains but not fulfilling the sCI criteria and ‘cognitively normal’ for the remaining patients.^2^

The cognitive tests were divided based on three different domains: memory (MEM), attention/information processing speed (ATT/IPS) and executive functions (EF). Patients were classified as impaired in each subdomain if they failed one or more tests in that domain.^31^

The emotional state was also evaluated using the Depression, Anxiety and Stress Scale-21 (DASS-21).^32^

### MRI acquisition

All patients were free from relapse and from steroid treatment for at least one month before they underwent the MRI examination. All brain images were acquired both at baseline (T0) and after two years (T2) using a 1.5 T scanner (Achieva, Philips Medical Systems, The Netherlands) with a 16-channel head coil.

The following MRI sequences were acquired:

- 2D double inversion recovery (DIR) sequence with 50 contiguous axial sections, repetition time [TR] = 15 631 ms, echo time [TE] = 25 ms, inversion time [TI] = 3400 ms, delay = 325 ms, slice thickness = 3.0 mm, number of averages = 2, voxel size = 0.97 × 0.97 mm^2^, matrix = 256 × 256 × 50, field of view (FOV) = 250 × 250 × 50 mm^3^, scanning time = 4.46 min;
- 2D T2 fluid-attenuated inversion recovery (FLAIR) with 50 contiguous axial sections, TR = 10 000 ms, TE = 120 ms, TI = 2500 ms, slice thickness = 3.0 mm, voxel size = 0.97 × 0.97 mm^2^, matrix = 256 × 256 × 50, FOV = 250 × 250 × 50 mm^3^, scanning time = 5.03 min;
- 3D fast field echo T1-weighted sequence with 120 axial sections, TR = 25 ms, TE = 4.6 ms, flip angle = 30°, slice thickness = 2.4 mm, in-slice spacing = 1.2 mm, voxel size = 0.97 × 0.97 mm^2^, matrix = 256 × 256 × 120, FOV = 250 × 250 × 145,2 mm^3^, scanning time = 5.18 min.

### MRI analysis

Since we aimed to investigate the predictive value of early brain volume loss, measured in the first two years from diagnosis, on long-term cognitive evolution, rather than considering baseline predictors measured at the time of diagnosis, we looked at the change in GM thickness/volume between T0 and T2. The longitudinal design was adopted using each subject as his/her control, to reduce the confounding effect of inter-individual morphological variability.^33^

Absolute regional measurements of the cortical and subcortical GM volumes and their relative changes after the first two years of multiple sclerosis (T0-T2) have been obtained by applying the Freesurfer^34^ image analysis suite (http://surfer.nmr.mgh.harvard.edu; release v5.3.0), a semi-automatic software based on two anatomical 3D T1-weighted Turbo Field Echo images acquired at each time point to increase the ratio signal-to-noise. A neurologist with decades of brain atrophy evaluation experience (M.C.), using a semi-automatic procedure WMLs segmentation and lesion filling procedure included in the Lesion Segmentation Tool for SPM, corrected topological defects in cortical surfaces due to WM lesions (WMLs).^35,36^ Briefly, WMLs were delineated on T2-weighted images using the intensity function and combined with previous segmented T1-weighted images for tissue classification. In case of overestimation of WMLs at the GM/WM boundary, a new intensity threshold was set. Data were completely anonymized to guarantee an analysis blinded to the clinical information. An averaged dataset was obtained for the T0 and one for the T2 scan. These two datasets were used in the longitudinal stream of the Freesurfer. Once aligned with surface-based registration methods, T0 and T2 masks were used to calculate the percentage of volume change for each hemisphere and for of each person with multiple sclerosis as follows: (T2 − T0)/T0. For each regions of interest of the Freesurfer parcellation, the averaged changes between left and right hemispheres were considered for the analyses.

T2-weighted images were utilized to account for supratentorial/infratentorial WM lesions. Considering the number of WMLs (WMLn), we identified four classes of patients (no lesions, 1–3 lesions, 4–9 lesions, ≥ 10 lesions) using a classification criterion proposed in a previous observational study.^37^

T2 FLAIR was the core sequence used for primary lesion identification. A confirmatory proton density/T_2_-weighted sequence was conducted in the posterior fossa, due to the sequence lower capability in lesion detection.^38^

The volume of the WMLs (WMLv) was calculated on T2 FLAIR images: a semi-automatic thresholding technique was conducted based on a fuzzy C-mean algorithm,^39^ included in the Medical Images Processing, Analysis and Visualization (http://mipav.cit.nih.gov) software developed at the National Institutes of Health.

The cortical lesion number (CLn) was evaluated on DIR images.^40^ In the present study, we mainly considered intracortical (within the cortex) and leukocortical lesions (within both the cortex and the underlying WM), due to the suboptimal performance of DIR sequences at 1.5 T field strengths in visualizing subpial lesions *in vivo*.^41,42^ Iuxtacortical lesions (lesions at the GM/WM junction adjacent to the cortex) that were identified on DIR images and confirmed on T2 FLAIR images were considered as WML and not combined with intracortical/leukocortical lesions; however, they have been counted together, considering them as a consequence of the evolution of the same neuropathological process over time.^43^ Moreover, cerebellar CL were also considered, since cortical demyelinating lesions are frequent in the cerebellar cortex.^44^

The volume of CLs (CLv) was calculated using a semi-automatic thresholding technique based on the fuzzy C-mean algorithm described above.^39^

### Statistical analyses

Statistical analyses were performed using R (https://www.r-project.org, version 3.5.3), STATA (StataCorp, College Station, TX, USA, version 18.0) and SPSS (SPSS Inc, Chicago, IL, USA, version 24) softwares.

Data distribution was tested through the Shapiro–Wilk test. As descriptive statistics, for quantitative data, mean ± SD or median [interquartile range (IQR)] were used; instead, for qualitative data, frequency and percentage were used.

The association between groups with different cognitive statuses and potential MRI determinants was investigated for categorical variables by chi-square test, *t*-test or Fisher’s exact test, and for continuous variables by analysis of covariance (ANCOVA), with Bonferroni correction, using age and disease duration as covariates.

Logistic regression models were conducted considering regional volume loss to evaluate its association with long-term global and domain-specific CI. Odds ratios (OR) were provided for each region, representing the risk of showing long-term CI. Brain regions that showed a significant result in the univariable logistic regression analyses were selected for a multivariable analysis to investigate the association between volume loss in each region and the global/domain-specific CI, in which adjustments were made for demographic/clinical variables (age, sex, disease duration) and volume of both WMLs and CLs. ORs were further provided for each region, representing the risk of showing long-term CI. The receiver operating characteristics (ROC) curve was calculated for each region. Significant brain regions in the univariable logistic regression analyses were selected for multivariable analyses, in which adjustment was made for age, sex, disease duration and volume of both WMLs and CLs. Bonferroni correction was performed. Power calculation was based on multivariable logistic regression using the response variable CI and global volume loss as independent variables. The model achieved 100% power.

The statistical significance level was set at 5%, while confidence intervals at 95%. A *P*-value of <0.05 was considered statistically significant.

## Results

### Patient characteristics

The sample comprised of 175 patients (118 females, 67%; mean ± SD age at the EOS = 47.7 ± 9.4 years).

At T0, after the baseline MRI, according to the diagnostic criteria of the time,^45^ 140 persons (80%) had RRMS and 35 persons (20%) had clinically isolated syndrome (CIS). At EOS, 127 persons (73%) had RRMS and 48 (27%) had SPMS.^18^

The median [IQR] EDSS^26^ at T0 was 1.0 [2.0], while at the EOS was 3.0 [3.5].

**Figure 1:**
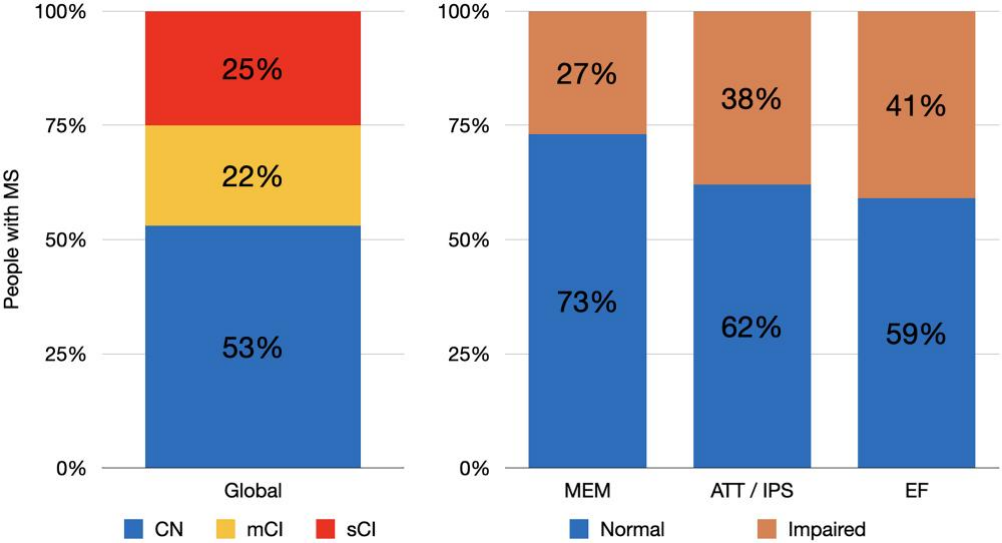
**Prevalence of global and domain cognitive status of people with multiple sclerosis.** Global and domain-specific cognitive characterization of multiple sclerosis sample after 20 years from diagnosis (*N* = 175). MS, multiple sclerosis; CN, cognitive normal; mCI, mild cognitive impaired; sCI, severe cognitive impaired; MEM, memory; ATT/IPS, attention/information processing speed; EF, executive functions.

At the EOS, 94 (53%) were CN, while 81 (47%) showed different levels of CI: 38 mCI (22%) and 43 sCI (25%). In particular, considering specific cognitive domains, 46 were impaired in MEM (27%), 66 were impaired in ATT/IPS (38%), and 71 were impaired in EF (41%) (Fig. 1).

Comparing demographic and clinical results among the three global cognitive groups (CN, mCI, sCI), ANCOVA showed a significant difference in age, disease duration and sex; therefore, we included them as covariates in the following analyses. Detailed demographic, clinical and MRI results of the whole sample, in addition to comparisons among CN, mCI and sCI groups, are provided in Table 1.

**Table 1:** Clinical and MRI results at diagnosis of the whole multiple sclerosis sample and separating individuals based on their cognitive status at the EOS

	Whole MS cohort (n = 175)	CN (n = 94)	mCI (n = 38)	sCI (n = 43)	CN versus mCI P	CN versus sCI P	mCI versus sCI P
Age at T0	29.8 ± 9.2	28.2 ± 8.8	30.0 ± 8.3	33.1 ± 9.8	n.s.	**0.009**	n.s.
Age at EOS	48.7 ± 9.4	46.0 ± 8.4	50.1 ± 9.1	53.2 ± 9.9	**0.049**	**<0.001**	n.s.
Sex F/M (%)	118/57 (67/33%)	74/20 (79/21%)	21/17 (55/45%)	23/20 (53/47%)	**0.006**	**0.003**	n.s.
Education	13.4 ± 3.7	14.3 ± 3.5	12.4 ± 3.2	12.5 ± 4.2	**0.020**	**0.026**	n.s.
CIS/RRMS at T0 (%)	35/140 (20/80%)	22/72 (23/77%)	8/30 (21/79%)	5/38 (12/88%)	n.s.	n.s.	n.s.
RR/SPMS at EOS (%)	127/48 (73/27%)	75/19 (80/20%)	26/12 (68/32%)	26/17 (60/40%)	n.s.	n.s.	n.s.
Disease duration at EOS	19.9 ± 5.1	18.9 ± 4.6	20.9 ± 5.0	21.2 ± 5.7	n.s.	**0.034**	n.s.
EDSS at T0	1.0 [2.0]	1.0 [2.0]	1.25 [2.0]	1.75 [2.5]	n.s.	**0.002**	n.s.
EDSS at EOS	3.0 [3.5]	2.0 [2.5]	3.0 [3.5]	5.0 [3.0]	n.s.	**<0.001**	n.s.
Global CTh at T0 (mm)	2.7 ± 0.3	2.8 ± 0.3	2.6 ± 0.3	2.5 ± 0.3	**0.026**	**<0.001**	n.s.
WMLn at T0	4–9	4–9	4–9	>10	n.s.	**0.002**	n.s.
WMLv at T0 (mm^3^)	2622 ± 2188	1935 ± 1826	3085 ± 2364	3713 ± 2249	**0.011**	**<0.001**	n.s.
New WMLn at T2	0 [1.0]	0 [1.0]	0 [1.25]	0 [1.0]	n.s.	n.s.	n.s.
CLn at T0	1.0 [4.0]	0 [3.0]	1.0 [3.25]	4.0 [7.0]	n.s.	**<0.001**	**<0.001**
CLv at T0 (mm^3^)	300 ± 423	181 ± 318	217 ± 298	634 ± 536	n.s.	**<0.001**	**<0.001**
New CLn at T2	0 [1.0]	0 [0]	0 [1.0]	0 [2.0]	n.s.	n.s.	n.s.
Cerebellar lesions at T0	0 [0]	0 [0]	0 [0]	0 [1.0]	n.s.	**0.002**	n.s.
Brainstem lesions at T0	0 [0]	0 [0]	0 [0]	0 [0]	n.s.	n.s.	n.s.

Mean ± SD was reported for continuous variables, while median [IQR] was reported for categorical variables. Significant (*P* < 0.05) values were reported in bold.

MS, multiple sclerosis; CN, cognitive normal; mCI, mild cognitive impaired; sCI, severe cognitive impaired; EOS, end of study; CIS, clinically isolated syndrome; RRMS, relapsing-remitting MS; SPMS, secondary progressive MS; EDSS, Expanded Disability Status Scale; CTh, cortical thickness; WMLn, white matter lesion number; WMLv, white matter lesion volume; CLn, cortical lesion number; CLv, cortical lesion volume.

No differences were found for DASS-21 between cognitive groups (*P* = 0.144).

### Regional MRI characterization of patients based on their cognitive profile

The median [IQR] time interval between MRI and diagnosis was 0 [2.0] years.

CI patients, independently from the domain impaired, showed more volume loss in precuneus (*P* < 0.001). Memory-impaired and attention/IPS-impaired patients showed a significantly higher volume loss in the hippocampus, insula, precentral gyrus, parahippocampal gyrus, cingulate gyrus, superior frontal gyrus and postcentral gyrus. In addition, attention/IPS-impaired patients showed a significantly higher volume loss in the cerebellum (Table 2).

**Table 2:** Regional volume loss results separating individuals based on their domain (memory, attention/IPS, executive functioning) cognitive status at the end of the study, ordered in terms of significance

	MEM N (n = 129)	MEM I (n = 46)	MEM N versus I P	ATT/IPS N (n = 109)	ATT/IPS I (n = 66)	ATT/IPS N versus I P	EF N (n = 104)	EF I (n = 71)	EF N versus I P
Precuneus	−1.1 ± 0.7%	−2.3 ± 1.1%	**<0.001**	−1.0 ± 0.5%	−2.1 ± 1.1%	**<0.001**	−1.2 ± 0.7%	−1.7 ± 1.2%	**<0.001**
Hippocampus	−1.5 ± 0.8%	−2.2 ± 1.1%	**<0.001**	−1.4 ± 0.7%	−2.1 ± 1.0%	**<0.001**	−1.5 ± 0.8%	−1.9 ± 1.0%	n.s.
Insula	−1.3 ± 0.7%	−2.2 ± 1.0%	**<0.001**	−1.3 ± 0.6%	−2.1 ± 1.0%	**<0.001**	−1.4 ± 0.7%	−1.8 ± 1.0%	n.s.
Precentral gyrus	−1.1 ± 0.5%	−1.5 ± 0.7%	**<0.001**	−1.0 ± 0.3%	−1.4 ± 0.8%	**<0.001**	−1.1 ± 0.4%	−1.4 ± 0.7%	n.s.
Parahippocampal gyrus	−1.6 ± 1.0%	−2.6 ± 1.0%	**<0.001**	−1.4 ± 0.9%	−2.6 ± 1.1%	**<0.001**	−1.6 ± 0.9%	−2.1 ± 1.2%	n.s.
Cingulate	−1.8 ± 1.0%	−2.9 ± 1.1%	**<0.001**	−1.7 ± 1.0%	−2.7 ± 1.1%	**<0.001**	−1.9 ± 1.2%	−2.2 ± 1.2%	n.s.
Superior frontal gyrus	−1.1 ± 0.4%	−1.6 ± 1.0%	**0.024**	−1.1 ± 0.4%	−1.5 ± 0.9%	**0.020**	−1.1 ± 0.5%	−1.4 ± 0.8%	n.s.
Postcentral gyrus	−1.1 ± 0.5%	−1.5 ± 0.8%	**0.048**	−1.0 ± 0.4%	−1.5 ± 0.8%	**<0.001**	−1.1 ± 0.4%	−1.3 ± 0.7%	n.s.
Cerebellum	−1.5 ± 1.1%	−2.2 ± 1.0%	n.s.	−1.3 ± 1.0%	−2.2 ± 1.1%	**<0.001**	−1.6 ± 1.1%	−1.7 ± 1.1%	n.s.
Thalamus	−1.6 ± 0.9%	−1.9 ± 0.7%	n.s.	−1.6 ± 0.8%	−1.9 ± 0.8%	n.s.	−1.7 ± 0.8%	−1.7 ± 0.9%	n.s.
Putamen	−0.9 ± 0.4%	−1.1 ± 0.3%	n.s.	−0.9 ± 0.4%	−1.1 ± 0.4%	n.s.	−1.0 ± 0.4%	−1.0 ± 0.4%	n.s.
Middle frontal gyrus	−0.9 ± 0.2%	−0.8 ± 0.2%	n.s.	−0.9 ± 0.2%	−0.9 ± 0.2%	n.s.	−0.9 ± 0.2%	−0.9 ± 0.3%	n.s.
Temporal pole	−1.0 ± 0.4%	−1.1 ± 0.4%	n.s.	−0.9 ± 0.3%	−1.0 ± 0.4%	n.s.	−1.0 ± 0.4%	−1.0 ± 0.4%	n.s.
Supplementary motor cortex	−0.9 ± 0.3%	−1.0 ± 0.4%	n.s.	−0.9 ± 0.3%	−1.0 ± 0.4%	n.s.	−1.0 ± 0.4%	−0.9 ± 0.3%	n.s.
Superior temporal gyrus	−0.9 ± 0.3%	−1.0 ± 0.4%	n.s.	−0.9 ± 0.3%	−1.0 ± 0.3%	n.s.	−1.0 ± 0.3%	−1.0 ± 0.3%	n.s.
Inferior temporal gyrus	−0.9 ± 0.3%	−0.9 ± 0.3%	n.s.	−0.9 ± 0.3%	−0.9 ± 0.3%	n.s.	−0.6 ± 0.2%	−0.6 ± 0.2%	n.s.
Middle temporal gyrus	−0.9 ± 0.2%	−0.9 ± 0.2%	n.s.	−0.9 ± 0.2%	−0.9 ± 0.2%	n.s.	−0.9 ± 0.2%	−0.9 ± 0.2%	n.s.
Amygdala	−0.9 ± 0.3%	−0.8 ± 0.3%	n.s.	−0.8 ± 0.3%	−0.9 ± 0.3%	n.s.	−0.8 ± 0.3%	−0.8 ± 0.2%	n.s.
Caudate	−0.7 ± 0.2%	−0.8 ± 0.3%	n.s.	−0.7 ± 0.2%	−0.8 ± 0.3%	n.s.	−0.8 ± 0.2%	−0.7 ± 0.3%	n.s.
Middle occipital gyrus	−0.7 ± 0.2%	−0.7 ± 0.2%	n.s.	−0.6 ± 0.2%	−0.7 ± 0.2%	n.s.	−0.6 ± 0.2%	−0.7 ± 0.2%	n.s.
Superior occipital gyrus	−0.7 ± 0.2%	−0.7 ± 0.2%	n.s.	−0.7 ± 0.2%	−0.7 ± 0.2%	n.s.	−0.7 ± 0.2%	−0.7 ± 0.2%	n.s.
Pallidum	−0.7 ± 0.2%	−0.7 ± 0.2%	n.s.	−0.7 ± 0.2%	−0.7 ± 0.2%	n.s.	−0.7 ± 0.2%	−0.7 ± 0.2%	n.s.
Cuneus	−0.6 ± 0.2%	−0.7 ± 0.1%	n.s.	−0.6 ± 0.2%	−0.6 ± 0.2%	n.s.	−0.6 ± 0.2%	−0.6 ± 0.2%	n.s.
Calcarine cortex	−0.6 ± 0.2%	−0.6 ± 0.2%	n.s.	−0.6 ± 0.2%	−0.6 ± 0.2%	n.s.	−0.6 ± 0.2%	−0.6 ± 0.2%	n.s.
Fusiform gyrus	−0.6 ± 0.2%	−0.6 ± 0.2%	n.s.	−0.6 ± 0.2%	−0.6 ± 0.2%	n.s.	−0.6 ± 0.2%	−0.6 ± 0.2%	n.s.
Gyrus rectus	−0.6 ± 0.2%	−0.6 ± 0.2%	n.s.	−0.6 ± 0.2%	−0.6 ± 0.2%	n.s.	−0.6 ± 0.2%	−0.6 ± 0.2%	n.s.
Inferior occipital gyrus	−0.6 ± 0.2%	−0.6 ± 0.2%	n.s.	−0.6 ± 0.2%	−0.6 ± 0.2%	n.s.	−0.6 ± 0.2%	−0.6 ± 0.2%	n.s.

Data represent relative change between T2 and T0 and are reported considering mean ± SD. *P*-values are adjusted for Bonferroni correction. Significant (*P* < 0.05) values were reported in bold.

MEM N, memory normal; MEM I, memory impaired; ATT/IPS N, attention/information processing speed normal; ATT/IPS I, attention/information processing speed impaired; EF N, executive functions normal; EF I, executive functions impaired.

### Logistic regression analysis and ROC curves

**Figure 2:**
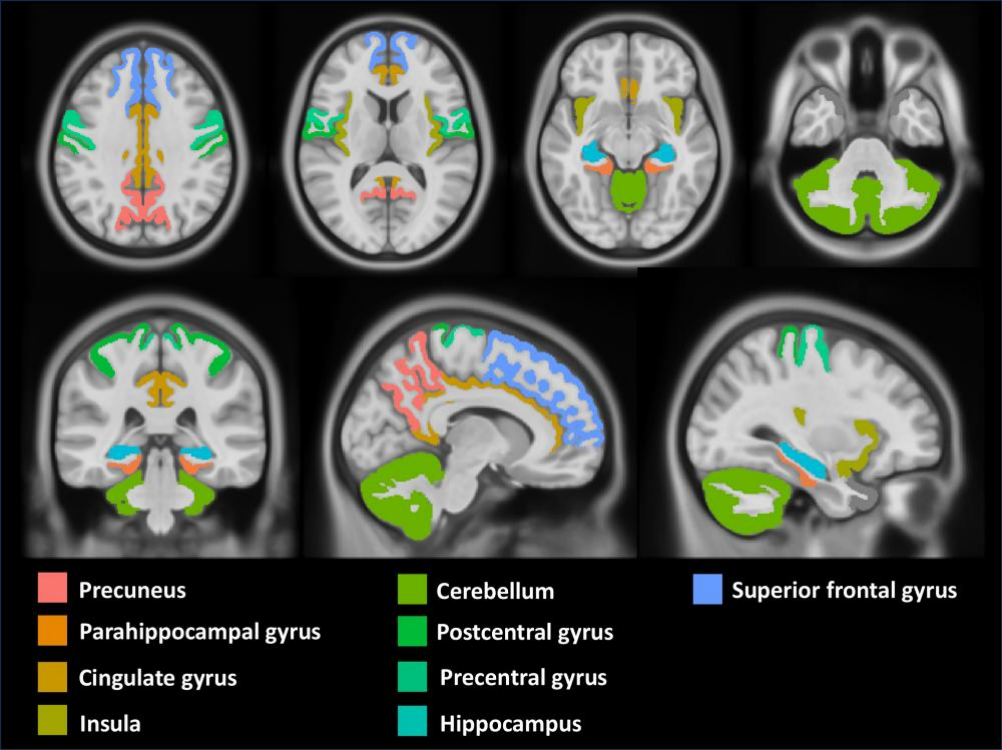
**Significant regional predictors for global cognitive status.** Illustration of the brain regions of interest (ROI) that showed statistical significance in the univariable logistic regression analyses (*P* < 0.05) with reference to global cognitive status.

**Figure 3:**
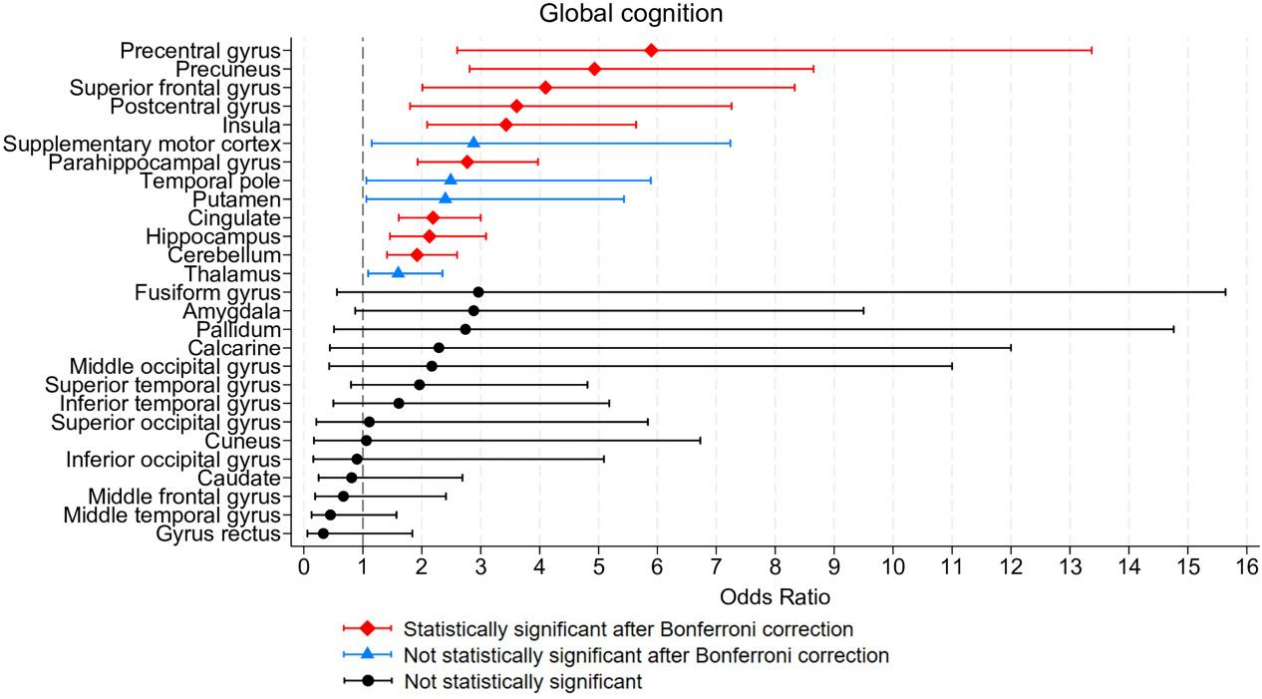
**Forest plot results for global cognitive status.** The forest plot illustrates the odds ratios (95% confidence intervals, CIs) derived from univariable logistic regression performed considering all multiple sclerosis patients (*N* = 175) for each cortical area associated with global cognitive status.

The GM volume loss in the precentral gyrus (OR = 5.90, *P* < 0.001), precuneus (OR = 4.92, *P* < 0.001), superior frontal gyrus (OR = 4.10, *P* < 0.001), postcentral gyrus (OR = 3.61, *P* < 0.001), insula (OR = 3.43, *P* < 0.001), parahippocampal gyrus (OR = 2.77, *P* < 0.001), cingulate (OR = 2.19, *P* < 0.001), hippocampus (OR = 2.13, *P* <0.001) and cerebellum (OR = 1.92, *P* < 0.001) during the first two years from the diagnosis was significantly associated with global CI at the EOS. Significant areas have been reported in Fig. 2. Detailed results are provided in Table 3 and Fig. 3.

**Table 3:** Association between percentage volume loss in brain regions between T0 and T2 and risk of global and domain CI at the EOS

Brain regions	Logistic regression		ROC curve	
	Odds ratio	*P*-value	Area under the curve	Empirical optimal cutpoint
Global cognitive status				
Precentral gyrus	5.90 (2.60–13.37)	**<0.001**	0.67 (0.58–0.76)	1.30
Precuneus	4.92 (2.81–8.65)	**<0.001**	0.79 (0.72–0.86)	1.27
Superior frontal gyrus	4.10 (2.01–8.33)	**<0.001**	0.67 (0.59–0.75)	1.14
Postcentral gyrus	3.61 (1.80–7.26)	**<0.001**	0.66 (0.58–0.74)	1.20
Insula	3.43 (2.09–5.64)	**<0.001**	0.73 (0.65–0.81)	1.09
Parahippocampal gyrus	2.77 (1.93–3.97)	**<0.001**	0.74 (0.66–0.82)	1.84
Cingulate	2.19 (1.61–2.99)	**<0.001**	0.75 (0.67–0.82)	1.56
Hippocampus	2.13 (1.46–3.09)	**<0.001**	0.67 (0.58–0.76)	1.34
Cerebellum	1.92 (1.41–2.60)	**<0.001**	0.69 (0.61–0.77)	1.45
MEM				
Precuneus	3.59 (2.34–5.50)	**<0.001**	0.82 (0.75–0.90)	1.31
Insula	3.32 (2.10–5.26)	**<0.001**	0.77 (0.69–0.85)	1.73
Precentral gyrus	3.28 (1.76–6.11)	**<0.001**	0.69 (0.59–0.78)	1.21
Superior frontal gyrus	3.25 (1.78–6.06)	**<0.001**	0.66 (0.57–0.76)	1.20
Postcentral gyrus	3.07 (1.64–5.75)	**0.027**	0.67 (0.58–0.76)	1.05
Parahippocampal gyrus	2.36 (1.69–3.30)	**<0.001**	0.77 (0.70–0.85)	1.78
Cingulate	2.30 (1.67–3.18)	**<0.001**	0.77 (0.70–0.85)	2.16
Hippocampus	2.17 (1.49–3.16)	**<0.001**	0.68 (0.59–0.78)	1.97
Cerebellum	1.90 (1.37–2.62)	**<0.001**	0.71 (0.63–0.79)	1.70
ATT/IPS				
Postcentral gyrus	4.90 (2.32–10.37)	**<0.001**	0.70 (0.62–0.78)	1.03
Precuneus	4.73 (2.80–7.87)	**<0.001**	0.82 (0.75–0.82)	1.10
Precentral gyrus	4.07 (2.04–8.09)	**<0.001**	0.63 (0.54–0.72)	1.31
Insula	3.43 (2.13–5.53)	**<0.001**	0.76 (0.69–0.83)	1.24
Parahippocampal gyrus	3.17 (2.19–4.59)	**<0.001**	0.81 (0.75–0.88)	1.78
Superior frontal gyrus	3.00 (1.63–5.51)	**<0.001**	0.62 (0.53–0.71)	1.20
Hippocampus	2.60 (1.75–3.85)	**<0.001**	0.74 (0.66–0.81)	1.28
Cingulate	2.36 (1.72–3.23)	**<0.001**	0.78 (0.71–0.85)	1.73
Cerebellum	2.33 (1.68–3.23)	**<0.001**	0.74 (0.67–0.82)	1.45
EF				
Precuneus	1.90 (1.35–2.67)	**<0.001**	0.65 (0.56–0.73)	0.94

95% confidence intervals (CIs) are reported for odds ratios and areas under the curve. *P*-values are adjusted for Bonferroni correction. Significant (*P* < 0.05) values were reported in bold.

EOS, end of study; ROC, receiver operating characteristics; MEM, memory; ATT/IPS, attention/information processing speed; EF, executive functions.

The GM volume loss in the precuneus (OR = 3.59, *P* < 0.001), insula (OR = 3.32, *P* < 0.001), precentral gyrus (OR = 3.28, *P* < 0.001), superior frontal gyrus (OR = 3.25, *P* < 0.001), postcentral gyrus (OR = 3.07, *P* = 0.027), parahippocampal gyrus (OR = 2.36, *P* < 0.001), cingulate (OR = 2.30, *P* < 0.001), hippocampus (OR = 2.17, *P* < 0.001) and cerebellum (OR = 1.90, *P* < 0.001) during the first two years from the diagnosis was significantly associated with memory impairment at the EOS.

The GM volume loss in the postcentral gyrus (OR = 4.90, *P* < 0.001), precuneus (OR = 4.73, *P* < 0.001), precentral gyrus (OR = 4.07, *P* < 0.001), insula (OR = 3.43, *P* < 0.001), parahippocampal gyrus (OR = 3.17, *P* < 0.001), superior frontal gyrus (OR = 3.00, *P* < 0.001), hippocampus (OR = 2.60, *P* < 0.001), cingulate (OR = 2.36, *P* < 0.001) and cerebellum (OR = 2.33, *P* < 0.001) during the first two years from the diagnosis was significantly associated with attention/IPS impairment at the EOS.

The GM volume loss in the precuneus (OR = 1.90, *P*<0.001) during the first two years from the diagnosis was significantly associated with executive impairment at the EOS.

**Figure 4:**
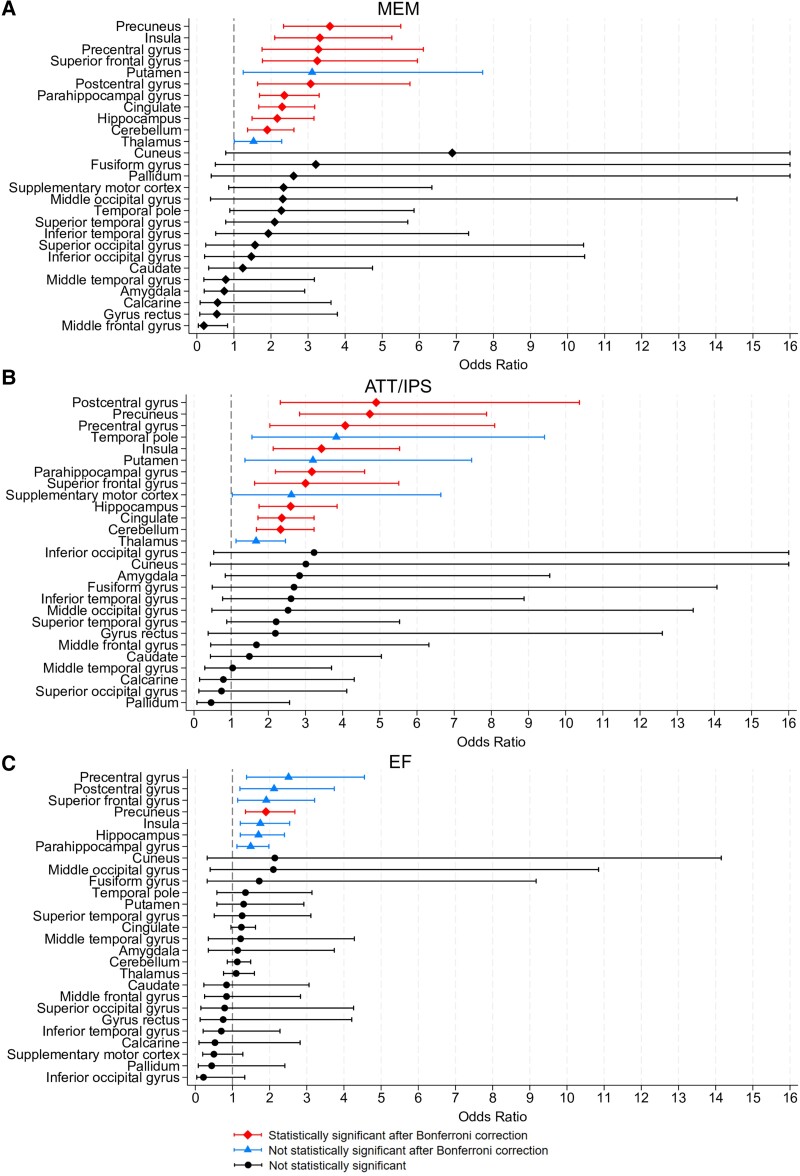
**Forest plot results for domain-specific cognitive status.** The forest plot illustrates the odds ratios (95% confidence intervals, CIs) derived from univariable logistic regression performed considering all multiple sclerosis patients (*N* = 175) for each cortical area associated with domain cognitive status (**A** = MEM, **B** = ATT/IPS, **C** = EF). To increase graph readability, the upper limit for the CIs of the odds ratios has been capped at 16. MEM, memory; ATT/IPS, attention/information processing speed; EF, executive functions.

Detailed results are provided in Table 3 and Fig. 4 (Fig. 4A for memory impairment; Fig. 4B for attention/IPS impairment; Fig. 4C for executive function impairment).

The ROC curve was also calculated, considering both global/domain CI, and an empirical optimal cutpoint along with the relative area under the curve was proposed for each region (Table 3).

Furthermore, ROC was calculated to show the predictive value of volume global loss, both independently and also adjusting for focal damage (accumulation of cortical lesions), with global cognitive status at the end of the study (Supplementary Fig. 1).

### Adjusted multivariable analysis

Regions that resulted in the logistic regression analysis were additionally investigated in a multivariable analysis adjusting for demographic/clinical (age, sex, disease duration) and for the volume of WMLs and CLs. We focused on lesion volumes because they have demonstrated their largest effect on multiple sclerosis-related cognitive impairment compared to total lesion number.^13,24^

GM volume loss in the precuneus (OR = 3.37, *P* < 0.001), insula (OR = 2.33, *P* = 0.036), parahippocampal gyrus (OR = 2.07, *P* < 0.001) and cingulate (OR = 1.81, *P* = 0.001) during the first two years from diagnosis was significantly associated with global CI after 20 years.

GM volume loss in the precuneus (OR = 3.00, *P* < 0.001), insula (OR = 2.35, *P* = 0.009), cingulate (OR = 1.94, *P* < 0.001) and parahippocampal gyrus (OR = 1.78, *P* = 0.027) during the first two years from diagnosis was significantly associated with memory impairment after 20 years.

GM volume loss in the precuneus (OR = 3.71, *P* < 0.001), parahippocampal gyrus (OR = 2.54, *P* < 0.001), insula (OR = 2.49, *P* = 0.009), cingulate (OR = 1.98, *P* < 0.001) and cerebellum (OR = 1.93, *P* < 0.001) during the first two years from diagnosis was significantly associated with attention/IPS impairment after 20 years. For the executive domain, multivariable analysis showed no significant results.

Detailed results are provided in Figs 3 and 4 and Table 4.

**Table 4:** Association between regional GM volume loss between T0 and T2 and risk of CI at the EOS, adjusted for age, sex, disease duration, and volumes of WM lesions and cortical lesions

Brain regions	Adjusted odds ratio	*P*-value
Global cognitive status		
Precuneus	3.37 (1.82–6.25)	**<0.001**
Insula	2.33 (1.31–4.14)	**0.036**
Parahippocampal gyrus	2.07 (1.37–3.13)	**<0.001**
Cingulate	1.81 (1.26–2.58)	**0.009**
Precentral gyrus	2.39 (0.90–6.39)	0.738
Postcentral gyrus	2.00 (0.87–4.56)	0.900
Superior frontal gyrus	1.78 (0.77–4.13)	1.000
Cerebellum	1.51 (1.05–2.16)	0.225
Hippocampus	1.30 (0.79–2.14)	1.000
MEM		
Precuneus	3.00 (1.78–5.05)	**<0.001**
Insula	2.35 (1.41–3.94)	**0.009**
Cingulate	1.94 (1.35–2.77)	**<0.001**
Parahippocampal gyrus	1.78 (1.21–2.63)	**0.027**
Superior frontal gyrus	1.72 (0.84–3.53)	1.000
Postcentral gyrus	1.66 (0.81–3.41)	1.000
Cerebellum	1.52 (1.06–2.19)	0.216
Hippocampus	1.43 (0.89–2.28)	1.000
Precentral gyrus	1.34 (0.60–2.96)	1.000
ATT/IPS		
Precuneus	3.71 (2.80–6.63)	**<0.001**
Parahippocampal gyrus	2.54 (1.69–3.81)	**<0.001**
Insula	2.49 (1.46–4.27)	**0.009**
Cingulate	1.98 (1.40–2.81)	**<0.001**
Cerebellum	1.93 (1.35–2.78)	**<0.001**
Postcentral gyrus	2.85 (1.26–6.46)	0.108
Hippocampus	1.87 (1.16–3.02)	0.108
Precentral gyrus	1.67 (0.72–3.83)	1.000
Superior frontal gyrus	1.40 (0.68–2.88)	1.000
EF		
Precuneus	1.37 (0.91–2.07)	0.131

95% confidence intervals (CIs) are reported for odds ratios. *P*-values are adjusted for Bonferroni correction. Significant (*P* < 0.05) values were reported in bold.

GM, grey matter; CI, cognitive impairment; EOS, end of study; WM, white matter; MEM, memory; ATT/IPS, attention/information processing speed; EF, executive functions.

## Discussion

We aimed to identify early MRI predictors, in terms of global and regional GM volume loss within the first two years from diagnosis, of long-term global and domain CI in people with multiple sclerosis. Results showed that patients who develop greater GM atrophy in eloquent areas within the first two years after diagnosis will suffer from more severe cognitive alterations after 20 years. The precuneus, insula, parahippocampal gyrus and cingulate were the best predictors of long-term global CI. Similar patterns were found for specific domain alterations, corroborating the role of parietal, insular, hippocampal, limbic and cerebellar regions.

The present results are in line with previous literature. At diagnosis and within the first two years of disease, people with multiple sclerosis show substantial brain damage, particularly involving cortex, cerebellum and deep GM nuclei:^46^ volume loss in specific brain regions drives the development of CI many years later.^47^ Previous works also reported that GM atrophy has been demonstrated as the first brain damage condition necessary for CI manifestation, and among brain structures going towards atrophy at first are the insula and hippocampus.^2^ In our results, precuneus is the region that showed the highest risk factor for a worse cognitive prognosis (adjusted OR for global CI = 3.37), both considering global impairment and specific cognitive domain alterations. Precuneus is highly involved in various complex functions, such as providing a connection to the hippocampus for parietal lobe visuospatial processes and related information (e.g. episodic memory). Its involvement has been reported in different neurodegenerative disorders,^48^ and its volumetric reduction sensitively highlights an underlying ongoing pathologic process.^49^ In people with relapse-onset multiple sclerosis, it has been demonstrated as one of the first areas to become atrophic, together with cingulate.^7^ Both structures are part of the posterior parietal cortex, working as the core of a widely interconnected and highly active cerebral network at rest and, therefore, more susceptible to metabolic/hypoxic damage, one of the significant pathological pathways of chronic tissue injury in multiple sclerosis.^50^ In particular, the cingulate gyrus, being one of the most demyelinated brain regions in people with multiple sclerosis,^51^ is also markedly subject to significant cortical atrophy via retrograde degeneration.^1^ The cingulate and hippocampus, along with the insula, are structures involved in several cognitive processes (e.g. attentive/executive and memory networks),^4,46^ and among the most affected regions by cortical pathology in multiple sclerosis:^7^ since they are surrounded by a reduced cerebrospinal fluid (CSF) flow, may be more subjected to chronic intrathecal inflammatory process due to a marked neuroaxonal irreversible damage.^1^ Early volume loss in the hippocampus, thalamus and cerebellum in people with multiple sclerosis within two years from diagnosis was associated with CSF inflammatory profile, considering their anatomical proximity to CSF boundaries and their conformation with deep folia limiting CSF flow.^52^ Cerebellar damage is typical in multiple sclerosis:^44^ neuropathological observations identify the cerebellar cortex as a typical site of demyelination.^53^ Due to its functional neural connections between the frontal and posterior parietal cortex and limbic circuit,^54^ the cerebellum plays a crucial role in cognition, particularly for attentive and memory functions.^55^Other cortical regions identified as predictors of long-term CI, such as parahippocampal gyrus, superior frontal gyrus, precentral gyrus and postcentral gyrus, corroborated previous studies showing their role in cognitive abilities.^5,56^

We acknowledge some limitations. Firstly, we did not have the chance to include a healthy cohort in the study, which would have been useful for a reference comparison for volumetric measures. Secondly, this single-centre investigation might have been influenced by selection bias. Considering the mild physical disability accrual after 20 years of multiple sclerosis, our inferences are mainly made on individuals characterized by favourable clinical course (‘benign multiple sclerosis’); however, cognitive deficits can be present in about half of persons with a low physical disability degree.^57^ Moreover, cognitive status was not associated to multiple sclerosis diagnosis at T0, otherwise at EOS, a significantly higher number of sCI were diagnosed with a secondary progressive multiple sclerosis course. This might represent an initial dissociation between physical and cognitive disability at the beginning of the disease, which over time tend to overlap, resulting in a global disability.^58^ Our results should be confirmed in larger and multi-centric studies. Furthermore, our region-wise analyses can only detect structural damage; future studies should also include functional MRI to understand the contribution of disruption to networks connecting different brain regions. Moreover, we did not consider different disease-modifying therapies during the multiple sclerosis course. Nonetheless, within the first two years of the disease, persons were treated with first-line drugs available at that time (around 20 years ago); in addition, so far, no approved medication is available for treating cognitive alterations in multiple sclerosis.^9,59^ Lastly, only for the ATT/IPS and the EF domains (not for MEM), around 10% of patients had missing data regarding neuropsychological test: however, this was limited to single tests, and also, for the majority of this patients, the classification of the cognitive impairment was not affected (they were already impaired in that domain, considering other tests administered).

Taken together, our data corroborated the association between cerebral GM damage and cognitive impairment, strengthening the hypothesis of higher susceptibility to neurodegenerative processes in key brain regions in multiple sclerosis. In particular, our results supported the relevance of specific grey matter regions of fronto-temporoparietal areas, cerebellum and deep grey matter since the time of diagnosis in predicting long-term cognitive alterations in people with multiple sclerosis.

## Supplementary Material

fcae355_Supplementary_Data

## Data Availability

The data that support the findings of this study are available from the corresponding author upon reasonable request. The accompanying code is provided as supplementary material in .do file format.
