# Supplementary material for: Early regional cerebral grey matter damage predicts long-term cognitive impairment phenotypes in multiple sclerosis: a 20-year study
Source: Brain Commun. 2024 Oct 12;6(6):fcae355. doi: 10.1093/braincomms/fcae355 (PMC11528517; doi:10.1093/braincomms/fcae355)
Supplement: fcae355_Supplementary_Data [file fcae355_supplementary_data.zip › Supplementary Figure 1.pdf]

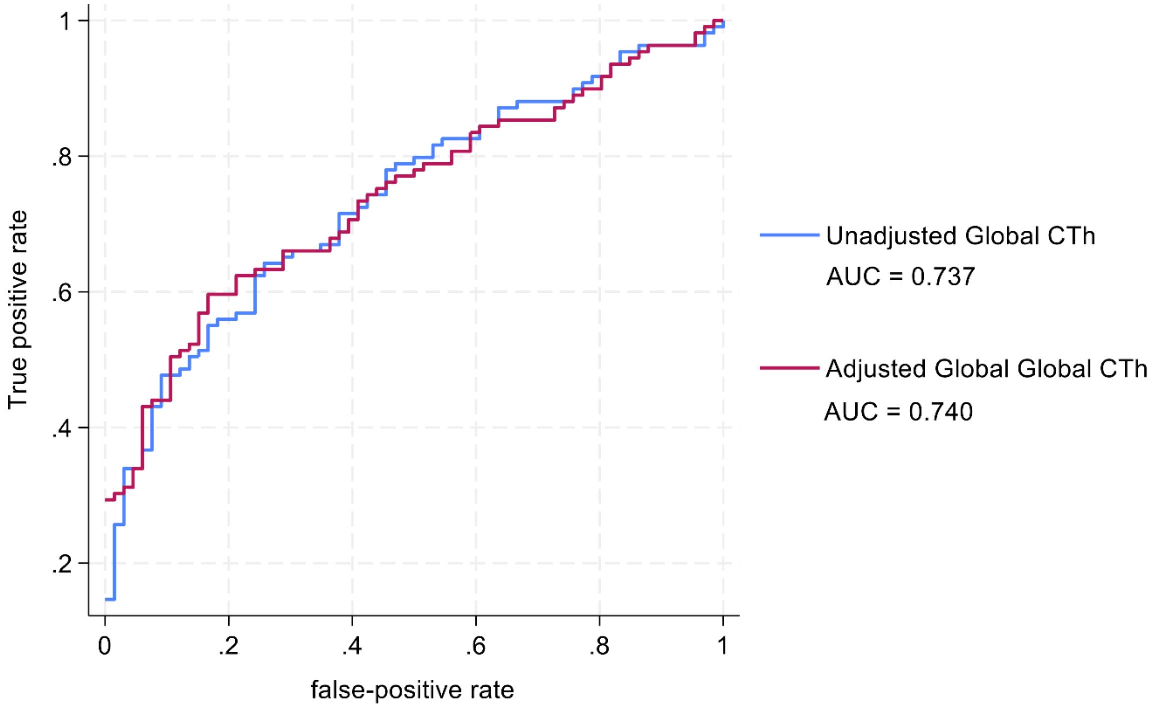

Supplementary Figure 1. Receiver operating characteristic (ROC) curve representing the capacity of global cortical thickness % loss to predict global cognitive impairment.

Adjusted curve uses as covariate number of new cortical lesions developed during the first two years of follow-up.

CTh=Cortical Thickness; AUC=Area Under the Curve
